# Self-reported acceptability and feasibility of a multimodal intervention to reduce antibiotic prescriptions for urinary tract infections in primary care: a process evaluation of the RedAres trial among general practitioners and medical practice assistants

**DOI:** 10.1186/s12913-025-13218-2

**Published:** 2025-08-30

**Authors:** Angela Schuster, Paula Tigges, Julianna Grune, Judith Kraft, Alexandra Greser, Ildikó Gágyor, Mandy Böhme, Anja Klingeberg, Tim Eckmanns, Andy Maun, Anja Mentzel, Guido Schmiemann, Christoph Heintze, Jutta Bleidorn

**Affiliations:** 1 Institute of General Practice and Family Medicine, Charité - Universitätsmedizin Berlin, 10117 Berlin, Germany; 2 Department of General Practice, University Hospital Wuerzburg, Josef- Schneider-Str. 2, D7, 97080 Wuerzburg, Germany; 3 Institute of General Practice, University Hospital Jena, 07743 Jena, Germany; 4 Robert Koch Institute, 13353 Berlin, Germany; 5 Institute of General Practice/Family Medicine, Faculty of Medicine and Medical Center, University of Freiburg, 79110 Freiburg im Breisgau, Germany; 6 Department of Health Services Research, Institute for Public Health and Nursing Research, University of Bremen, 28359 Bremen, Germany

**Keywords:** Antimicrobial resistance, Urinary tract infection, Primary care, General practitioners, Practice staff, Process evaluation, Acceptability, Antibiotic stewardship, Multimodal intervention, Prescribing behaviour

## Abstract

**Background:**

Urinary tract infections are common and lead to frequent and inappropriate antibiotic prescribing in primary care. The RedAres randomized controlled trial has shown to reduce second-line antibiotic use for urinary tract infections through a multimodal intervention. It included guideline recommendations for general practitioners and patients (1), provision of regional data for antibiotic resistance (2), delivering personalized feedback based on the proportion of first- and second-line antibiotic prescriptions (3), and benchmarking with regional or supra-regional practices (4). To discuss all interventions, individual telephone counselling was offered. The aim of the RedAres process evaluation is to assess the acceptability of the multimodal intervention among general practitioners and the feasibility of the study design for medical practice assistants.

**Methods:**

The general practitioners and medical practice assistants surveys were conducted following the RedAres intervention during the last trial visit. To assess acceptability among general practitioners, we developed a questionnaire based on Sekhon´s theoretical framework of acceptability. The questionnaires for medical practice assistants included questions on data management feasibility and attitudes towards this task. Data were analyzed using SPSS and R. Cases were weighted according to the number of respondents per practice, descriptive statistics, chi-squared tests, bivariable logistic regressions, and multivariable logistic regressions were used for data analysis.

**Results:**

The response rate to the questionnaires was 96.6% for general practitioners (*N* = 63) and 91.5% for medical practice assistants (*N* = 56). Most general practitioners (93.9%) found the multimodal intervention to be adequate for enhancing guideline adherence. Among the intervention components, resistance data (31.8%) and prescribing feedback (31.8%) were equally appreciated, while benchmarking (12.2%) was the least appreciated intervention. Most medical practice assistants (96.3%) reported being satisfied with the data collection, documentation, and transfer in the RedAres study.

**Conclusions:**

The RedAres intervention was favorably received by general practitioners, medical practice assistants deemed data management and extraction feasible. With the backdrop of the intervention’s effectiveness, its favorable reception, and its practicability, antibiotic stewardship and quality control measures implemented at the practice level hold promise for effectively enhancing guideline adherence and improving antibiotic stewardship practices in real-world settings.

**Trial registration:**

Prospective registration at the German Clinical Trial Register (DRKS), trial number DRKS00020389, registration date 30.01.2020 (https//drks.de/search/en/trial/DRKS00020389).

**Supplementary Information:**

The online version contains supplementary material available at 10.1186/s12913-025-13218-2.

## Background

Urinary tract infections (UTIs) are a prevalent health concern, affecting 9 to11% of women in the annually [1, 2]. Despite clinical guidelines recommending symptomatic treatment, delayed prescription or narrow-spectrum antibiotics for uncomplicated UTIs [3, 4], the use of broad-spectrum antibiotics remains a concern [5–7] and is associated with an increase in resistance patterns [8].

General practitioners (GPs) adherence to clinical guidelines for diagnosing and treating UTIs is low and inadequate antibiotic prescriptions are prevalent [9, 10]. GPs poor adherence to guidelines may be related to safety concerns, logistic barriers, knowledge gaps, the lack of diagnostic possibilites, unclear clinical presentations [11, 12] and the wish for treatment autonomy [13].

To increase guideline adherence for UTIs, various strategies have been developed, but the most effective strategy remains to be identified [14]. Multimodal interventions, including training, prescription feedback, information on local resistance patterns and prescribing feedback based on individual and peer prescription behavior (benchmarking) have shown to be effective in different ambulatory care settings [15–20]. However, knowledge on acceptability and scalability of antibiotic stewardship interventions in general practice is scarce [21]. Understanding the factors that determine physicians acceptance of interventions to curb antibiotic overuse is crucial for effective control of antimicrobial resistance (AMR) [22].

The RedAres multicenter cluster randomized trial (RCT) demonstrated the effectiveness of a multimodal intervention in reducing antibiotic prescriptions for uncomplicated urinary tract infections in women. The intervention significantly decreased the proportion of second-line antibiotics by a mean of −0.13 (95% CI: −0.21 to −0.06, *P* < 0.001), corresponding to a relative reduction of 40%, after adjusting for preintervention proportions. This comprehensive approach included information materials on the clinical guideline for managing UTIs (I), information on regional resistance data (II), feedback on guideline-aligned prescribing (III), and benchmarking of GPs’ prescribing behavior compared to peers (IV). For further questions or support, telephone counseling was offered by from experienced peers (researchers with GP qualifications) [23, 24]. Data analysis was conducted on aggregated prescription data extracted from electronic medical records (EMR) by medical practice assistants (MPA) at practice level. MPAs, who are versatile healthcare professionals combining patient care, medical assistance, and administrative duties, were chosen for this task due to their existing data management skills. By using aggregated data, we ensured efficient data collection without overburdening the GPs, as we did not need to include patients at individual level. The process evaluation of the RedAres study aims to understand GPs’ perspectives on the acceptability of interventions and to evaluate their potential for implementation into routine care. Furthermore, we aim to evaluate the feasibility of routine data handling from the perspective of the MPA involved in the study.

Methods.

The RedAres process evaluation is an integral part of the RedAres trial. This evaluation included in-depth interviews with GPs to collaboratively develop intervention materials [25] and qualitatively assess their perspectives on the interventions [26]. The intervention materials included guideline recommendations for general practitioners and patients, information on regional antibiotic resistance data [27], quarterly feedback including individual first line and second line antibiotic prescribing, bench-marking with regional or national peers, and telephone counselling [24].

The current analysis is based on a cross-sectional survey conducted among GPs and MPA in 57 intervention practices across four German regions (Berlin – Brandenburg, Bavaria, Baden-Württemberg, and Thuringia) following the intervention phase of the RedAres trial. All GPs and MPA involved in the intervention arm of the RCT were invited to participate. GPs and MPA who were unable to complete the survey during the last visit were asked to send the survey via mail.

The RedAres study was registered on the 30.01.2020 under the trial registration number DRKS00020389 at the Trial registration site DRKS, ethics approval was obtained at the Ethics Committee of the Medical Faculty, University of Wuerzburg in November 2019 (Number: 20191106 01).

To assess the acceptance of the intervention by GPs we adapted the theoretical framework of acceptability (TFA) by Sekhon et al. [28] and developed a survey for GPs with 20 questions to assess the dimensions of self-efficacy (I), affective attitude (II), and perceived effectiveness (III) for each of the four interventions. Additionally, we assessed information gain (IV) for information on UTI management and resistance data.

Self-efficacy (I) was operationalized as the GPs confidence in their ability to use the intervention materials effectively during consultations, affective attitude (II) as participants’ comfort level with the intervention, perceived effectiveness (III) as the degree to which they believed the intervention material would promote rational antibiotic prescribing and information gain (IV) as the likelihood to acquire knowledge from the intervention material. The GPs responded to acceptability statements on a five-point Likert scale, with responses ranging from strong agreement to strong disagreement. The survey with MPA included 16 questions to assess the feasibility of extracting, documenting, and transmitting aggregated patient data from the EMR into study documentation, as well as motivators and learnings during the study. Where applicable, ease to perform a task was assessed using four-point Likert scale ranging from very easy to very difficult. GPs and MPA questionnaires are displayed in the supplements 1 till 4.

Survey data were transferred from paper to SPSS Version 29.0, followed by data cleaning for duplicates, implausible and missing values, and formatting errors. Descriptive statistics were performed in SPSS, and regression analysis was performed in R Version 4.2.2. Cases were weighted according to the number of respondents per practice.

Continuous variables were plotted as histograms and boxplots to visualize their distribution and assess outliers. All other variables were visualized in stratified cross tables.

We assessed approval rates for each of the intervention’s four dimensions and overall approval among GPs. For overall approval we dichotomized the responses by considering “approval” and “strong approval” as approval, and all other responses as non-approval. To simplify data analysis, we combined “strong disagreement” and “disagreement” into a single category of disagreement for each intervention’s dimensions I-IV.

The association between approval and sociodemographic or practice-related characteristics was calculated using the chi-squared test. The effect size was estimated using Phi (φ), and the significance threshold was set at *p* < 0.05.

To assess the association between GPs self-reported dimensions of acceptability for each intervention with GPs’ age we performed a bivariable logistic regression for each intervention (dependent variable) and calculated crude odds ratios (cOR) as well as 95% confidence intervals (95% CI). After testing for model assumptions, we calculated multivariable logistic regressions, to control for confounding we adjusted for the following individual and practice related covariates: federal state, gender, position in practice, working hours, size of city of the practice, type of practice (single or multiple owned), training practice and number of patient visits per quartile. For each multivariable logistic regression, we calculated adjusted odds ratios (aOR) and 95% CI, as well as their respective p-value with level of significance set at *p* < 0.05. Due to the low sample size and respectively large confidence intervals, we refrain from reporting OR and 95% and indicate significant difference only in a semi-quantitative manner.

For MPA, we calculated the overall satisfaction rate with the study task and for the following components: filter data in the EMR, extract data from EMR, aggregate extracted patient data, document anonymized data in the study data form and enter data in the electronic research database of the study. We also assessed motivators for study participation, MPA learning experiences, and the ability to integrate learned competencies into everyday practice.

## Results

We surveyed 119 health professionals from 59 intervention practices, 63 were GPs form 57 intervention practices (response rate per practice 96.6%), and 56 were MPA from 54 intervention practices (response rate per practice 91.5%).

To account for multiple responses from a practice we weighed each questionnaire based on the number of replies per practice.

### Acceptability of the RedAres trial among general practitioners

In Table 1 we display the stratified and weighted sociodemographic characteristics of GPs participating in the process evaluation of the RedAres study (see Table 1), for practice characteristic see supplement 5. Provenance across the different regions and gender was well-balanced. Among our cohort of GPs only 21.6% were under 40 years old, which accurately reflects the aging population of GPs. Most GPs owned the practice where they worked.

**Table 1:** Stratified sociodemographic characteristics of gps participating in the RedAres study

*n*/*N* *N* = 63*	*n* (%)*	Gender M (*n* (%))/ W (*n* (%))	Age group < 42 (*n* (%))/43–52 (*n* (%))/ 53–62 (*n* (%))/>63 (*n* (%))	Region BER/BB(*n* (%))/BW (*n* (%))/ BY (*n* (%))/TH (*n* (%))	Position in practice Self-employed (*n* (%))/ employed (*n* (%))	Working hours Fulltime (*n* (%))/ parttime (*n* (%))	Years of professional experience < 5 (*n* (%))/6–15 (*n* (%))/ > 15 (*n* (%))
Gender (57/63)							
Male (m)	29 (51)		6 (21)/8 (29)/ 11 (39)/3 (11)	7 (24)/7 (24)/ 12 (41)/3 (10)	24 (83)/ 5 (17)	26 (90)/ 3 (10)	4 (14)/7 (24)/ 18 (62)
Female (f)	28 (49)		7 (25)/9 (32)/ 9 (33)/3 (11)	8 (29)/6 (21)/ 7 (25)/7 (25)	24 (86)/ 4 (14)	21 (75)/ 7 (25)	2 (7)/12 (41)/ 15 (52)
Age group (55/63)							
< 42 (n, %)	12 (22)	6 (46)/ 7 (54)		4 (33)/5 (42)/ 2 (17)/1 (8)	7 (54)/ 6 (46)	8 (61)/ 5 (38)	6 (46)/7 (54)/ 0 (0)
43–52 (n, %)	17 (30)	8 (47)/ 9 (53)		5 (30)/1 (6)/ 6 (35)/5 (30)	14 (82)/ 3 (18)	15 (88)/ 2 (12)	0 (0)/11 (62)/ 7 (39)
53–62 (n, %)	20 (36)	11 (55)/ 9 (45)		4 (20)/6 (30)/ 7 (35)/3 (15)	20 (100)/ 0 (0)	17 (85)/ 3 (15)	0 (0.)/0 (0.)/ 20 (100.)
> 63 (n, %)	6 (11)	3 (50)/ 3 (50)		1 (17)/1 (17)/ 4 (67)/0 (0)	6 (100)/ 0 (0)	6 (100)/ 0 (0)	0 (0)/0 (0)/ 6 (100)
Region (57/63)							
Berlin/Brandenburg	15 (26)	7 (47)/ 8 (53)	4(29)/5(36)/ 4(29)/1(7)		11(73)/ 4 (27)	13 (88)/2 (12)	3 (20)/6 (40)/6 (40)
Baden-Württemberg	13 (23)	7 (54)/ 6 (46)	5(38)/1(8)/ 6(46)/1(8)		11(85)/ 2 (15)	11 (84)/2 (15)	2 (15)/3 (23)/8 (61)
Bayern	19 (33)	12 (63)/ 7(37)	2(10)/6(32)/ 7(37)/4(22)		19(100)/ 0 (0)	16 (84)/3 (16)	0 (0)/6 (32)/13 (69)
Thüringen	10 (18)	3 (30)/ 7(70)	1(11)/5(55)/ 3(33)/0(0.0)		7 (70)/ 3 (3)	7 (70)/3 (30)	1 (5)/4 (35)/6 (60)
Position in practice (57/63)							
Self-employed	48 (84)	24 (50)/ 24 (50)	7 (15)/14 (30)/ 20 (43)/6 (13)	11 (23)/11 (23)/ 19 (40)/7 (15)		44 (912)/ 4 (8)	1 (2)/ 14 (29)/33 (69)
Employed (n, %)	9 (15)	5 (56)/ 4 (44)	6 (67)/3 (33)/ 0 (0)/0 (0)	4 (44)/2 (22)/ 0 (0)/3 (33)		3 (33)/ 6 (67)	5 (56)/ 4 (44)/0 (0)

Weighted according to replays per practice (*n* = 57)

Most GPs (93.9%, *N* = 53) found the multimodal intervention to be adequate for enhancing guideline adherence. The most helpful intervention components were information on regional resistance data and feedback on prescribing behavior, each cited as most useful by 31.8% of participants. Only 12.2% considered benchmarking the most useful intervention.

Nearly half of the GPs (48.5%) considered digital delivery of information materials and feedback to be the most effective communication channel. However, 39.2% preferred printed information and 12.3% opted for a combination of print and digital information.

**Fig. 1:**
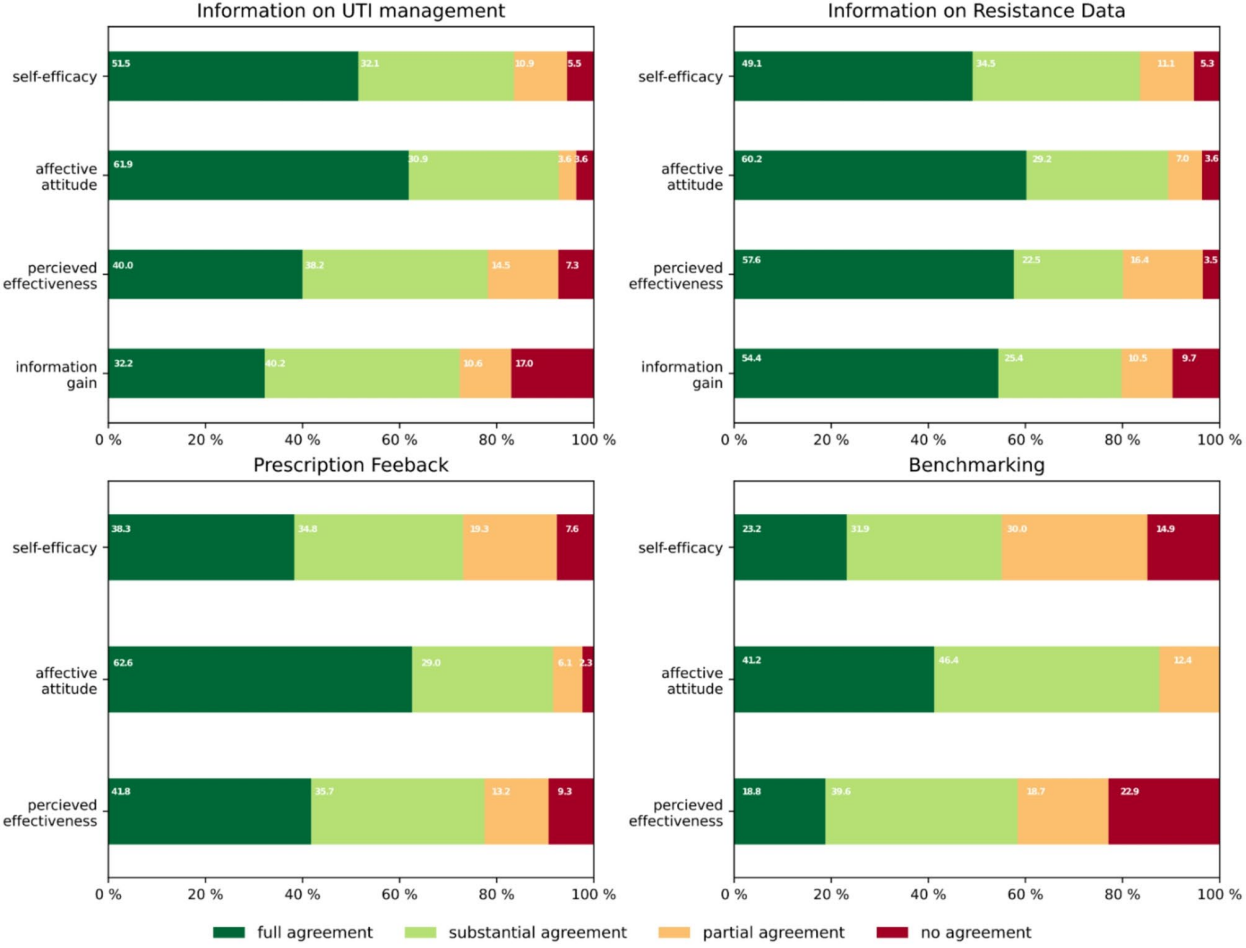
GPs agreement with the dimensions of acceptability for each intervention in the RedAres study

Figure 1 illustrates the extent to which GPs perceived the interventions of the RedAres study as acceptable in its different dimensions. Participating GPs expressed positive affective attitudes towards all interventions, with agreement towards the individual interventions ranging between 89 and 93%. Compared to the other interventions, GPs indicated relatively lower agreement with prescription feedback and benchmarking regarding self-efficacy (59%) and perceived effectiveness (53%), while affective attitude was comparable. Agreement towards self-efficacy, perceived effectiveness, affective attitude, and information gain was highest for information on resistance patterns, followed by information material on UTI guidelines. These findings align with data from qualitative interviews, which revealed that GPs were interested in the resistance situation and in regional variations and used this information to opt for less-resistant first-line treatment options [26]. However, in our qualitative study some GPs raised concerns about the comparability and usefulness of benchmarking [26].

After controlling for potential confounding factors, we found significantly higher agreement among GPs in Baden-Württemberg or Thuringia (BW or TH) compared to Bavaria (BY) regarding the perceived effectiveness of prescription feedback and its likelihood to influence their individual prescribing practices. In addition, we found that healthcare providers under the age of 40 were more likely than their older colleagues to believe that feedback on prescriptions and information material on resistance data had a positive impact on their prescribing behavior.

Moreover, male GPs expressed greater confidence in the positive influence of prescription feedback on their prescribing practices in daily practice.

### Practice staff assessment of data management within the RedAres trial

The sociodemographic analysis of the MPA survey revealed that 98% of all MPA were female, with 26% of all MPA coming from Berlin, 22.2% from Baden-Württemberg, 31.5% from Bavaria, and 20.4% from Thuringia. In Table 2 we display the stratified and weighted sociodemographic characteristics of our MPA cohort (see Table 2).

**Table 2:** Stratified sociodemographic characteristics of MPA participating in the RedAres study

(*n*/*N*) *N* = 56*	*N* (%)	Gender f (*n*, %)/m (*n*,%)	federal state BER (*n*, %)/BW (*n*, %)/ BY (*n*, %)/TH (*n*, %)	Training for PA Yes (*n*, %)/No (*n*, %)	Years of professional experience < 5 (*n*, %)/5.1–14.9 (*n*, %)/>15 (*n*, %)	Working hours minor (*n*,%)/ parttime (*n*,%)/ Fulltime t (*n*,%)
Gender (48/56)						
Female (f)	47 (98)		12 (25)/11 (23)/ 16 (34)/8 (17)	45 (96)/2 (4)	10 (22)/13(29)/22 (49)	2 (4)/12 (25)/33 (70)
Male (m)	1 (2)		1 (100)/0 (0)/ 0 (0)/0 (0)	1 (100)/0 (0)	1 (100)/0 (0)/0 (0)	0 (0)/0 (0)/1 (100)
Federal state (54/56)						
Berlin (BER)	14 (26)	12 (92)/1 (8)		12 (92)/1 (8)	2 (15)/5 (38)/6 (46)	1 (7)/1 (7)/12 (86)
Baden-Württemberg (BW)	12 (22)	11 (100)/0 (0)		11 (100)/0 (0)	2 (18)/5 (45)/4 (36)	1 (8)/5 (42)/6 (50)
Bayern (BY)	17 (31)	16 (100)/0 (0)		16 (100)/0 (0)	3 (20)/2 (13)/10 (66)	0 (0)/7 (44)/9 (56)
Thüringen (TH)	11 (20)	8 (100)/0 (0)		7 (87)/1 (12)	4 (57)/1 (14)/2 (29)	0 (0)/0 (0)/8 (100)
Training for PA (48/56)						
Yes	46 (96)	45 (98)/1 (2)	12 (26)/11 (23)/16 (34)/7 (15)		10 (23)/12 (27)/22 (50)	2 (4)/12 (26)/32 (70)
No	2 (4)	2 (100)/0 (0)	1 (50)/0 (0)/ 0 (0)/1 (50)		1 (50)/1 (50)/0 (0)	0 (0)/0 (0)/2 (100)
Years of professional experience (46/56)						
< 5 years	11 (24)	10 (90.9)/1 (9.1)	2 (18)/2 (18)/3 (27)/4 (36)	10 (91)/1 (9)		0 (0)/1 (9.)/10 (90)
5.1–14.9 years	13 (27)	13 (100)/0 (0)	5 (38)/5 (38)/2 (15)/1 (8)	12 (92)/1 (8)		1 (8)/4 (31)/8 (61)
> 15 years	22 (48)	22 (100)/0 (0)	6 (27)/4 (18)/10 (45)/2 (9)	22 (100)/0 (0)		1 (4)/6 (27)/15 (69)
Working hours (48/56)						
Minor	2 (4)	2 (100)/0 (0)	1 (50)/1 (50)/ 0 (0)/0 (0)	2 (100)/0 (0)	0 (0)/1 (50)/1 (50)	
Parttime	12 (25)	12 (100)/0 (0)	1 (8)/5 (38)/ 7 (52)/0 (0%)	12 (100)/0 (0)	1 (9.)/4 (36)/6 (54)	
Fulltime	34 (71)	33 (97)/1 (3)	12 (34)/6 (17)/ 9 (26)/8 (23)	32 (94)/2 (6)	10 (30)/8 (24)/15 (45)	

Weighted according to replays per practice (*N* = 54)

Possible deviation from 100% are due to rounding errors

**Fig. 2:**
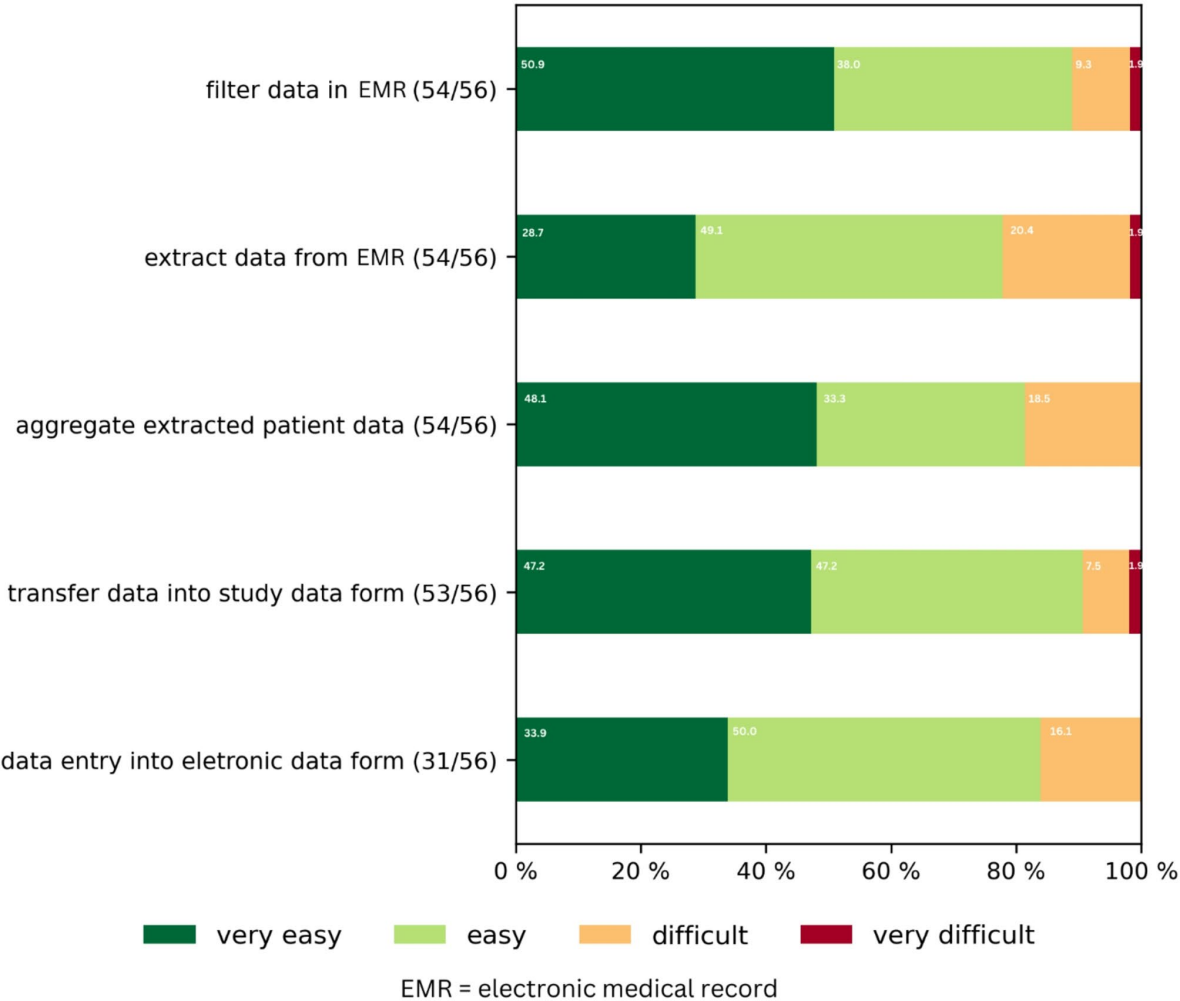
MPA assessment of the single data extraction and management study tasks

The great majority of MPA (96.3%) reported being satisfied with the data collection, documentation, and transfer in the RedAres study and most considered the single data processing tasks as easy or very easy (see Fig. 2). However, only 31 out of 56 MPA entered aggregated data into the electronic research database RedCap which might indicate a rather high threshold to perform that task. When MPAs were unable to perform this task, researchers themselves filled in the data. For most MPA motivation to participate to RedAres was triggered by the possibility to be part of a research project (56.5%) or to learn new competences (50.0%). 38% of the MPA were motivated by the change in daily working routines, financial incentives were mentioned by nearly one third of all MPA (31.5%).

The majority of MPA (78.3%) lacked prior experience with research projects. However, 77.4% of MPA felt that their competencies in research data management and EMR use within the practice had improved. Additionally, 96.2% of MPA expressed confidence in their ability to collect and manage data in their practice. However, most MPA reported not to use those competences in daily practice (81.3%), further a big share of MPA considered that newly acquired competences were not sufficiently appreciated in the practice teams (61.5%).

## Discussion

### Principal findings

The implementation of the multimodal RedAres intervention resulted in a significant decrease in both the proportion of second-line antibiotics prescribed and the overall number of antibiotic prescriptions for uncomplicated UTIs in women treated in primary care settings [24]. While the precise contribution of each individual intervention remains unclear [24], the process evaluation indicated that all four interventions were favorably perceived by participating GPs, suggesting their potential for integration into routine clinical practice.

Overall, merely informative interventions such as information on UTI management and resistance data went along with higher agreement, while interventions associated with evaluation and judgment of performance agreement was lower. Benchmarking was the least appreciated intervention. Although this format is considered one of the most effective interventions for quality improvement in health care [29, 30]; reluctance towards benchmarking aligns with the results of our qualitative study with the same cohort [26] and previous qualitative studies [13] and can be attributed to concerns regarding its validity, resource constraints, entrenched resistance to change, and the specific challenges associated with implementing new practices [31].This hesitancy is further compounded by the complex nature of clinical decision-making in primary care settings, where patient expectations and the pressure to prescribe antibiotics can conflict with stewardship goals [32]. Additionally, GPs may feel that benchmarking oversimplifies the nuanced process of antibiotic prescribing, potentially leading to a one-size-fits-all approach that doesn’t account for individual patient needs or local antimicrobial resistance patterns [26].

Conversely, information on prescription feedback was the most appreciated intervention. This is likely due to its innovation potential, as readily available information on regional resistance patterns stemming from ambulatory care for GPs is lacking [24], even though guidelines recommend orientation on such information [33].

While the affective attitude dimension exhibited the highest level of agreement, the possibility of social desirability bias, a common phenomenon when assessing acceptability, should be considered [34, 35].

MPA demonstrated a strong interest in participating in research projects, confidently assessing their competence in processing patient data. This assessment aligns with interim quality control evaluations conducted throughout the randomised controlled trial [24]. Despite the perceived feasibility of entering aggregated data into the electronic research database, nearly half of the MPAs refrained from performing this task. This indicates that future research should adopt more user-friendly data management tools to boost participation and data collection efficiency. To emulate the success of antibiotic stewardship interventions and monitoring practices, as commonly implemented in Scandinavia [36] and the UK [37, 38], Germany requires the swift development of data safe and automated solutions for the extraction of routine prescription data from EMRs.

Previous studies have established a positive association between MPA job satisfaction and opportunities for professional development [39]. Given the limited human resources in healthcare, particularly in the ambulatory sector [40, 41], incorporating data management tasks into the job profiles of MPA could serve as a valuable tool for attracting and retaining MPA within GP practices. However, for sustainable job satisfaction, MPA need recognition for their engagement [39, 42] which our cohort appears to lack.

While financial incentives have been shown to increase research participation rates [43], in our study only one-third of participants mentioned financial motivation as a relevant factor for their involvement. This is surprising as previous studies have identified dissatisfaction about financial remuneration among MPA [39, 42].

Currently, MPA extract specific information from the EMR at the end of each quarter for accounting purposes with the statutory health insurance. However, this data is not routinely utilized for quality improvement measures, such as reducing antibiotic prescriptions, while our qualitative interviews revealed that GPs support this task-sharing approach with their MPA for antibiotic stewardship.

Drawing upon Hawes et al.‘s scoping review on evidence-based measures to enhance antibiotic stewardship in primary care, implementing an interprofessional approach that leverages EMR data for quality control by MPA supported by automated data extraction algorithms could foster interprofessional collaboration and promote adherence to guidelines [21].

### Acceptability of antibiotic stewardship interventions

Interventions not directly targeting prescribers, such as modifying antibiotic package sizes, have demonstrated higher acceptability compared to those directly affecting GPs [44]. The importance of prescriber autonomy appears to be context-dependent and plays a significant role in Germany [13]. In line with these findings, nudge interventions, which subtly alter the decision-making process without restricting autonomy or imposing penalties, have demonstrated effectiveness in various settings [29]. The RedAres RCT exemplifies this approach, as GPs were given the opportunity to discuss their personalized feedback in a person-centered manner via phone calls with a senior GP researcher. However, the acceptability of nudging interventions does not automatically correlate with their effectiveness [45]. Gerstel has shown that acceptability of nudging appears to be contingent on pre-existing autonomous motivation, while self-regulatory capacity seems to play a less important role [46]. In our cohort, data format incompatibilities prevented us from directly matching prescribing performance with intervention acceptability. Based on Gerstel’s findings [46], we postulate that acceptability might have been highest among high performers. However, our RCT revealed that our interventions were most beneficial among low performers [24]. Effectively reaching low-performing target groups while preserving high levels of intervention acceptability may necessitate a trade-off that warrants further scrutiny during research and implementation. Artificial intelligence based knowledge support systems to improve antibiotic prescribing are acceptable among primary care providers [47] and might be also of use in identifying adequate target groups for antibiotic stewardship interventions [48].

Financial incentives in antibiotic stewardship have demonstrated effectiveness, particularly when penalties are implemented [49]. However, evidence on their acceptability among healthcare workers remains scarce and debatable [50, 51]. General populations acceptability for health promotion interventions is higher when incentives are offered rather than penalties [50, 52, 53]. Among primary care providers in Great Britain, pay-for-performance schemes have been implemented and are acceptable [54]. The high acceptability of our intervention might be attributed to the absence of financial penalties.

Interestingly in our study acceptability of antibiotic stewardship interventions did not reveal any substantial variations between eastern Germany (Berlin/Brandenburg and Thuringia) and western Germany (Baden-Württemberg). However, recent claims database analysis indicates higher antibiotic prescription rates in western Germany compared to eastern Germany among adults [55]. Consistent with previous research [56] demonstrating prolonged, and potentially guideline-deviant, antibiotic prescribing patterns among older general practitioners (GPs), our cohort analysis revealed lower acceptability among healthcare providers aged > 40 years. Interestingly, male GPs exhibited higher confidence in the positive impact of prescription feedback on their prescribing practices and professional self-efficacy, contrasting with previous findings showing a greater awareness for AMR among female GPs [57]. Our results might be attributed to a gender gap in self-efficacy which has been observed among GPs [58] and other specialists [59].

### Generalizability and practical implementation

Our results contribute to a growing body of evidence showing that multimodal interventions for antibiotic stewardship yield mixed and diverse outcomes across different studies.

Hartmann et al. demonstrated that multimodal interventions, including peer discussion rounds, benchmarking, and informational material on antibiotic prescribing, can promote higher rates of guideline-adherent prescribing among elder populations [60], similar results have been shown by De Vries et al. in Cape Town [61]. However, Aghlmandi and colleagues were unable to show improvement in antibiotic prescribing for respiratory and urinary tract infections through audit and feedback with peer benchmarking [62].

Vellinga et al. multimodal intervention led to an increase in first-line antibiotic prescriptions for urinary tract infections. However, this improvement in prescribing quality was accompanied by an unintended overall increase in antibiotic prescribing rates [20]. These contrasting findings highlight the complexity of implementing interventions to improve antibiotic prescribing practices and underscore the need for careful consideration of study design and potential unintended consequences.

The RedAres study’s intervention components, including guideline information, quarterly prescription feedback with regional benchmarking, and regional resistance data, could be integrated into the current healthcare system. The implementability of these components is demonstrated by existing practices of the Association of Statutory Health Insurance Physicians, which already uses benchmarking and feedback mechanisms to promote cost efficient prescribing through drug target reports comparing GP prescribing patterns [13].

However, with regard to feasibility a key challenge remains the extraction of aggregated antibiotic prescribing data from EMR, which was done manually by the MPAs in the RedAres study. Widespread adoption would be facilitated by developing secure technical solutions for extracting and transmitting aggregated patient data. This would streamline the process and make it more feasible for large-scale implementation, enhancing the potential for improving antibiotic stewardship in primary care settings. To achieve a sustainable impact, regular feedback on antibiotic prescribing is essential due to the dynamic and ever-changing nature of antibiotic resistance patterns. Additionally, repeated interventions have been shown to improve the efficacy of antibiotic adherence [63]. To enhance feasibility, integrating our multimodal intervention into clinical decision support systems—ideally within existing EMR—could facilitate implementation while keeping costs low [64].

### Strengths and limitations

Consistent findings across multiple German regions, stable and sustained implementation of the intervention throughout the study period [24], and the integration of quantitative and qualitative data all contribute significantly to strengthening the internal validity of our study.

However, we did not employ triangulation, which may limit the depth of our findings, as relying on a single methodological approach increases the risk of bias and may result in a less comprehensive understanding of the research topic.

Social desirability is a well know phenomenon when it comes to acceptability of guideline adherence [65] and cannot be excluded in our cohort. While the high response rate suggests that selection bias may not have had a significant impact on the study’s findings, the possibility that GPs with more favorable attitudes towards the intervention were more likely to participate cannot be entirely ruled out.

The study’s inclusion of GPs from diverse regions across Germany broadens its geographic representation, allowing for a more comprehensive understanding of antibiotic prescribing practices across the country. However, the studies representativeness is questionable as GPs with a particular interest in rational antibiotic prescribing might have been overrepresented. Linking the intervention’s acceptability to prescription patterns was not feasible due to data format incompatibilities, this limited our ability to identify potential discrepancies. To minimize the burden on GPs during the last monitoring visit of the RedAres RCT, our acceptability questionnaire was designed to be concise. We focused the dimensions affective attitude, self-efficacy, and perceived effectiveness of Sekhon´s TFA [28] and also assessed knowledge gain for interventions. Sekhon´s acceptability dimensions ethical considerations, intervention coherence, burden, and opportunity costs were not explicitly addressed [28]. Nevertheless, a comprehensive analysis encompassing various dimensions of acceptability was conducted in the accompanying qualitative paper [26].

### Conclusions and recommendations for practice

The RedAres trial indicates that, in addition to being effective in reducing second-line antibiotic prescribing, the intervention was also considered acceptable by GPs in our cohort, suggesting its potential as a promising approach for implementation. Person-centered nudging interventions and those that focus on information convergence may be more favorably received compared to interventions that directly evaluate or judge GPs’ performance. Data extraction from EMR and processing for research purposes by MPA was deemed feasible in our study, suggesting that integrating MPA into quality control measures for antibiotic prescribing at the practice level could support GPs to enhance their guideline adherence.

Our study adds to the emerging body of evidence on the acceptability of antibiotic stewardship interventions and underscores the potential value of assessing acceptability in both research and implementation contexts.

## Supplementary Information

Below is the link to the electronic supplementary material.

Supplementary Material 1

Supplementary Material 2

Supplementary Material 3

Supplementary Material 4

Supplementary Material 5

## Data Availability

The data set analysed in this study is available on request from the corresponding author. The data are not publicly available due to data safety restrictions agreed with the interviewees.

## Abbreviations

AMR: Antimicrobial resistance
GP: General practitioner
EMR: Electronic medical records
MPA: Medical practice assistants
RCT: Randomised controlled trial
RedAres: REDuction of Antibiotic RESistance trail.
TFA: Theoretical framework of acceptability

## References

[1] Butler CC, Hawking MKD, Quigley A, McNulty CAM. Incidence, severity, help seeking, and management of uncomplicated urinary tract infection: a population-based survey. Br J Gen Pract J R Coll Gen Pract. 2015;65:e702–707. 10.3399/bjgp15X686965 PMC4582883 26412847

[2] Dichieva S. (2015) Urinary tract infections in women (Harnwegsinfekte bei Frauen). In: Glaeske G, Schicktanz C, editors. Barmer GEK Arzneimittel-Report 2015. Schriftenreihe zur Gesundheitsanalysen. Wuppertal: Barmer GEK, 2015. pp.107–37.

[3] Deusche Gesellschaft für Urologie e.V. S3 Leitlinie Epidemiologie, Diagnostik, Therapie, Prävention und Management unkomplizierter, bakterieller, ambulant erworbener Harnwegsinfektionen bei Erwachsenen (HWI).

[4] National Institute for Health and Care Excellence. NICE guideline: urinary tract infection. antimicrobial prescribing; 2018. 39937934

[5] Butler CC, Francis N, Thomas-Jones E, et al. Variations in presentation, management, and patient outcomes of urinary tract infection: a prospective four-country primary care observational cohort study. Br J Gen Pract. 2017;67:e830–41. 29158245 10.3399/bjgp17X693641 PMC5697553

[6] Gágyor I, Strube-Plaschke S, Rentzsch K, Himmel W. Management of urinary tract infections: what do Doctors recommend and patients do? An observational study in German primary care. BMC Infect Dis. 2020;20:813. 33167875 10.1186/s12879-020-05377-w PMC7650164

[7] Petersen I, Hayward AC. Antibacterial prescribing in primary care. J Antimicrob Chemother. 2007;60:i43–7. 17656380 10.1093/jac/dkm156

[8] European Centre for Disease Prevention and Control. (2022) Antimicrobial resistance surveillance in Europe 2022 – 2020 data. https://www.ecdc.europa.eu/en/publications-data/antimicrobial-resistance-surveillance-europe-2022-2020-data. Accessed 18 Dec 2023.

[9] Lindbäck H, Lindbäck J, Melhus Å. Inadequate adherence to Swedish guidelines for uncomplicated lower urinary tract infections among adults in general practice. APMIS. 2017;125:816–21. 28585332 10.1111/apm.12718

[10] McIsaac WJ, Low DE, Biringer A, Pimlott N, Evans M, Glazier R. The impact of empirical management of acute cystitis on unnecessary antibiotic use. Arch Intern Med. 2002;162:600–5. 11871930 10.1001/archinte.162.5.600

[11] Harbin NJ, Lindbæk M, Romøren M. Barriers and facilitators of appropriate antibiotic use in primary care institutions after an antibiotic quality improvement program – a nested qualitative study. BMC Geriatr. 2022;22:458. 35624423 10.1186/s12877-022-03161-w PMC9137170

[12] Hartman EAR, Groen WG, Heltveit-Olsen SR, et al. Decisions on antibiotic prescribing for suspected urinary tract infections in frail older adults: a qualitative study in four European countries. Age Ageing. 2022;51:afac134. 35697352 10.1093/ageing/afac134 PMC9191618

[13] Mentzel A, Maun A. Ambulantes verordnungsverhalten von antibiotika und einstellung zum verordnungsfeedback. Z Für Allg. 2023;99:21–7.

[14] Arnold SR, Straus SE. Interventions to improve antibiotic prescribing practices in ambulatory care. Cochrane Database Syst Rev. 2005. 10.1002/14651858.CD003539.pub2. 16235325 10.1002/14651858.CD003539.pub2 PMC7003679

[15] Butler CC, Simpson SA, Dunstan F, et al. Effectiveness of multifaceted educational programme to reduce antibiotic dispensing in primary care: practice based randomised controlled trial. BMJ. 2012;344:d8173. 22302780 10.1136/bmj.d8173 PMC3270575

[16] Chauhan LR, Huang M, Abdo M, Church S, Fixen D, MaWhinney S, Miller M, Erlandson KM. Impact of a pilot multimodal intervention to decrease antibiotic use for respiratory infections in a geriatric clinic. Antimicrob Steward Healthc Epidemiol. 2022;2:e1. 36310812 10.1017/ash.2021.238 PMC9614947

[17] Hallsworth M, Chadborn T, Sallis A, Sanders M, Berry D, Greaves F, Clements L, Davies SC. Provision of social norm feedback to high prescribers of antibiotics in general practice: a pragmatic National randomised controlled trial. Lancet. 2016;387:1743–52. 26898856 10.1016/S0140-6736(16)00215-4 PMC4842844

[18] Kuehlein T, Goetz K, Laux G, Gutscher A, Szecsenyi J, Joos S. Antibiotics in urinary-tract infections. Sustained change in prescribing habits by practice test and self-reflection: a mixed methods before-after study. BMJ Qual Saf. 2011;20:522–6. 21262789 10.1136/bmjqs.2010.047357

[19] Samore MH, Bateman K, Alder SC, et al. Clinical decision support and appropriateness of antimicrobial prescribing: A randomized trial. JAMA. 2005;294:2305. 16278358 10.1001/jama.294.18.2305

[20] Vellinga A, Galvin S, Duane S, Callan A, Bennett K, Cormican M, Domegan C, Murphy AW. Intervention to improve the quality of antimicrobial prescribing for urinary tract infection: a cluster randomized trial. Can Med Assoc J. 2016;188:108–15. 26573754 10.1503/cmaj.150601 PMC4732960

[21] Hawes L, Buising K, Mazza D. Antimicrobial stewardship in general practice: A scoping review of the component parts. Antibiotics. 2020;9:498. 32784918 10.3390/antibiotics9080498 PMC7459857

[22] Sirota M, Habersaat KB, Betsch C et al. (2023) We must Harness the power of social and behavioural science against the growing pandemic of antimicrobial resistance. Nat Hum Behav 1–3. 10.1038/s41562-023-01762-y 37985918

[23] Gágyor I, Greser A, Heuschmann P, et al. REDuction of antibiotic resistance (REDARES) in urinary tract infections using treatments according to National clinical guidelines: study protocol for a pragmatic randomized controlled trial with a multimodal intervention in primary care. BMC Infect Dis. 2021;21:990. 34556027 10.1186/s12879-021-06660-0 PMC8461906

[24] Schmiemann G, Greser A, Maun A, et al. Effects of a multimodal intervention in primary care to reduce second line antibiotic prescriptions for urinary tract infections in women: parallel, cluster randomised, controlled trial. BMJ. 2023;383:e076305. 37918836 10.1136/bmj-2023-076305 PMC10620739

[25] Petruschke I, Stichling K, Greser A, Gagyor I, Bleidorn J. The general practitioner perspective of a multimodal intervention for the adequate use of antibiotics in urinary tract infection – a qualitative interview study. Z Für Evidenz Fortbild Qual Im Gesundheitswesen. 2022;170:1–6. 10.1016/j.zefq.2021.12.012 35283054

[26] Schuster A, Tigges P, Grune J, et al. GPs’ perspective on a multimodal intervention to enhance Guideline-Adherence in uncomplicated urinary tract infections: A qualitative process evaluation of the multicentric RedAres Cluster-Randomised controlled trial. Antibiotics. 2023;12:1657. 38136690 10.3390/antibiotics12121657 PMC10740691

[27] Klingeberg A, Willrich N, Schneider M, Schmiemann G, Gágyor I, Richter D, Noll I, Eckmanns T. The percentage of antibiotic resistance in uncomplicated Community-Acquired urinary tract infections. Dtsch Arzteblatt Int. 2024;121:175–81. 10.3238/arztebl.m2023.0267 PMC11079811 38221865

[28] Sekhon M, Cartwright M, Francis JJ. Acceptability of healthcare interventions: an overview of reviews and development of a theoretical framework. BMC Health Serv Res. 2017;17:88. 28126032 10.1186/s12913-017-2031-8 PMC5267473

[29] Raban MZ, Gonzalez G, Nguyen AD, Newell BR, Li L, Seaman KL, Westbrook JI. Nudge interventions to reduce unnecessary antibiotic prescribing in primary care: a systematic review. BMJ Open. 2023;13:e062688. 36657758 10.1136/bmjopen-2022-062688 PMC9853249

[30] Willmington C, Belardi P, Murante AM, Vainieri M. The contribution of benchmarking to quality improvement in healthcare. A systematic literature review. BMC Health Serv Res. 2022;22:139. 35109824 10.1186/s12913-022-07467-8 PMC8812166

[31] Williams J, Brown C, Springer A. Overcoming benchmarking reluctance: a literature review. Benchmarking Int J. 2012;19:255–76.

[32] Simpson SA, Wood F, Butler CC. General practitioners’ perceptions of antimicrobial resistance: a qualitative study. J Antimicrob Chemother. 2007;59:292–6. 17110392 10.1093/jac/dkl467

[33] Schmiemann G, Gebhardt K, Hummers-Pradier E. (2018) Burning on micturition (Brennen beim Wasserlassen) guideline of the German College of General Practitioner and Family Physicians DEGAM, 2018.

[34] Kramer L, Rabanizada N, Haasenritter J, Bösner S, Baum E, Donner-Banzhoff N. Do guidelines on first impression make sense? Implementation of a chest pain guideline in primary care: a systematic evaluation of acceptance and feasibility. BMC Fam Pract. 2011;12:128. 22103603 10.1186/1471-2296-12-128 PMC3267789

[35] Kulczycki A, Qu H, Shewchuk R. Primary care physicians’ adherence to guidelines and their likelihood to prescribe the human papillomavirus vaccine for 11- and 12-Year-Old girls. Womens Health Issues Off Publ Jacobs Inst Womens Health. 2016;26:34–9. 10.1016/j.whi.2015.07.012 PMC4690766 26344447

[36] Holm A, Cordoba G, Aabenhus R. Prescription of antibiotics for urinary tract infection in general practice in Denmark. Scand J Prim Health Care. 2019;37:83–9. 30689491 10.1080/02813432.2019.1569425 PMC6452818

[37] McNulty C, Hawking M, Lecky D, Jones L, Owens R, Charlett A, Butler C, Moore P, Francis N. Effects of primary care antimicrobial stewardship outreach on antibiotic use by general practice staff: pragmatic randomized controlled trial of the TARGET antibiotics workshop. J Antimicrob Chemother. 2018;73:1423–32. 29514268 10.1093/jac/dky004 PMC5909634

[38] Smith S, Hawker JI, Smith GE, Morbey R, Johnson AP, Fleming DM, Shallcross L, Hayward AC. A standardized methodology for the surveillance of antimicrobial prescribing linked to clinical indications in primary care. J Public Health Oxf Engl. 2018;40:630–8. 10.1093/pubmed/fdx114 PMC6166589 28977493

[39] Mergenthal K, Güthlin C. Predictors of job satisfaction among health care assistants. Z Für Evidenz Fortbild Qual Im Gesundheitswesen. 2021;167:78–85. 10.1016/j.zefq.2021.09.006 34815195

[40] Bundesagentur für Arbeit. (2022) Engpassanalyse - Statistik der Bundesagentur für Arbeit. https://statistik.arbeitsagentur.de/DE/Navigation/Statistiken/Interaktive-Statistiken/Fachkraeftebedarf/Engpassanalyse-Nav.html

[41] Wallenfels M. Damoklesschwert Fachkräftemangel. Wo sollen die künftigen MFA herkommen?; 2023.

[42] Gavartina A, Zaroti S, Szecsenyi J, Miksch A, Ose D, Campbell SM, Goetz K. Practice assistants in primary care in Germany – associations with organizational attributes on job satisfaction. BMC Fam Pract. 2013;14:110. 23915225 10.1186/1471-2296-14-110 PMC3750430

[43] Abdelazeem B, Abbas KS, Amin MA, El-Shahat NA, Malik B, Kalantary A, Eltobgy M. The effectiveness of incentives for research participation: A systematic review and meta-analysis of randomized controlled trials. PLoS ONE. 2022;17:e0267534. 35452488 10.1371/journal.pone.0267534 PMC9032371

[44] Mauffrey V, Kivits J, Pulcini C, Boivin JM. Perception of acceptable antibiotic stewardship strategies in outpatient settings. Médecine Mal Infect. 2016;46:285–93. 10.1016/j.medmal.2016.06.006 27475666

[45] Barbaroux A, Benoit L, Raymondie RA, Milhabet I. Nudging health care workers towards a flu shot: reminders are accepted but not necessarily effective. A randomized controlled study among residents in general practice in France. Fam Pract. 2021;38:410–5. 33506858 10.1093/fampra/cmab001

[46] van Gestel LC, Adriaanse MA, de Ridder DTD. Who accepts Nudges? Nudge acceptability from a self-regulation perspective. PLoS ONE. 2021;16:e0260531. 34860843 10.1371/journal.pone.0260531 PMC8641879

[47] Hurley R, Jury F, van Staa TP, Palin V, Armitage CJ. Clinician acceptability of an antibiotic prescribing knowledge support system for primary care: a mixed-method evaluation of features and context. BMC Health Serv Res. 2023;23:367. 37060063 10.1186/s12913-023-09239-4 PMC10103677

[48] Wathne JS, Skodvin B, Charani E, Harthug S, Blix HS, Nilsen RM, Kleppe LKS, Vukovic M, Smith I. Identifying targets for antibiotic stewardship interventions through analysis of the antibiotic prescribing process in hospitals - a multicentre observational cohort study. Antimicrob Resist Infect Control. 2020;9:114. 32693826 10.1186/s13756-020-00749-y PMC7374853

[49] Yoshikawa Y, Feldhaus I, Özçelik E, Hashiguchi TCO, Cecchini M. Financial strategies targeting healthcare providers to promote the prudent use of antibiotics: a systematic review of the evidence. Int J Antimicrob Agents. 2021;58:106446. 34610457 10.1016/j.ijantimicag.2021.106446

[50] Hoskins K, Ulrich CM, Shinnick J, Buttenheim AM. Acceptability of financial incentives for health-related behavior change: an updated systematic review. Prev Med. 2019;126:105762. 31271816 10.1016/j.ypmed.2019.105762

[51] Zetts RM, Stoesz A, Garcia AM, Doctor JN, Gerber JS, Linder JA, Hyun DY. Primary care physicians’ attitudes and perceptions towards antibiotic resistance and outpatient antibiotic stewardship in the USA: a qualitative study. BMJ Open. 2020;10:e034983. 32665343 10.1136/bmjopen-2019-034983 PMC7365421

[52] Giles EL, Sniehotta FF, McColl E, Adams J. Acceptability of financial incentives for health behaviour change to public health policymakers: a qualitative study. BMC Public Health. 2016;16:989. 27633661 10.1186/s12889-016-3646-0 PMC5025536

[53] Promberger M, Brown RCH, Ashcroft RE, Marteau TM. Acceptability of financial incentives to improve health outcomes in UK and US samples. J Med Ethics. 2011;37:682–7. 21670321 10.1136/jme.2010.039347 PMC3198007

[54] Lester H, Matharu T, Mohammed MA, Lester D, Foskett-Tharby R. Implementation of pay for performance in primary care: a qualitative study 8 years after introduction. Br J Gen Pract. 2013;63:e408–15. 23735412 10.3399/bjgp13X668203 PMC3662458

[55] Scholle O, Asendorf M, Buck C, Grill S, Jones C, Kollhorst B, Riedel O, Schüz B, Haug U. Regional variations in outpatient antibiotic prescribing in germany: A small area analysis based on claims data. Antibiotics. 2022;11:836. 35884090 10.3390/antibiotics11070836 PMC9312140

[56] Fernandez-Lazaro CI, Brown KA, Langford BJ, Daneman N, Garber G, Schwartz KL. Late-career physicians prescribe longer courses of antibiotics. Clin Infect Dis Off Publ Infect Dis Soc Am. 2019;69:1467–75. 10.1093/cid/ciy1130 30615108

[57] Saha SK, Kong DCM, Thursky K, Mazza D. A nationwide survey of Australian general practitioners on antimicrobial stewardship: awareness, uptake, collaboration with pharmacists and improvement strategies. Antibiotics. 2020;9:310. 32521720 10.3390/antibiotics9060310 PMC7345044

[58] Murray MA, Cardwell C, Donnelly M. GPs’ mental wellbeing and psychological resources: a cross-sectional survey. Br J Gen Pract. 2017;67:e547. 28716997 10.3399/bjgp17X691709 PMC5519126

[59] Meister T, Foessleitner P, Breuer G, Winder FM, Favero M, Friemann M, Krischer B, Weiss M, Windsperger K. The impact of gender on the self-confidence of practical and surgical skills among OBGYN residents: a trinational survey. Arch Gynecol Obstet. 2023. 10.1007/s00404-023-07202-6. 37695372 10.1007/s00404-023-07202-6 PMC11147860

[60] Hartman EAR, Pol AC, van de, Heltveit-Olsen SR, et al. Effect of a multifaceted antibiotic stewardship intervention to improve antibiotic prescribing for suspected urinary tract infections in frail older adults (ImpresU): pragmatic cluster randomised controlled trial in four European countries. BMJ. 2023;380:e072319. 36813284 10.1136/bmj-2022-072319 PMC9943914

[61] De Vries E, Johnson Y, Willems B, Bedeker W, Ras T, Coetzee R, Tembo Y, Brink A. Improving primary care antimicrobial stewardship by implementing a peer audit and feedback intervention in cape town community healthcare centres. South Afr Med J Suid-Afr Tydskr Vir Geneeskd. 2022;112:812–8. 10.7196/SAMJ.2022.v112i10.16397 36472332

[62] Aghlmandi S, Halbeisen FS, Saccilotto R, et al. Effect of antibiotic prescription audit and feedback on antibiotic prescribing in primary care: A randomized clinical trial. JAMA Intern Med. 2023;183:213–20. 36745412 10.1001/jamainternmed.2022.6529 PMC9989898

[63] Schwartz KL, Xu AXT, Alderson S, et al. Best practice guidance for antibiotic audit and feedback interventions in primary care: a modified Delphi study from the joint programming initiative on antimicrobial resistance: primary care antibiotic audit and feedback network (JPIAMR-PAAN). Antimicrob Resist Infect Control. 2023;12:72. 37516892 10.1186/s13756-023-01279-z PMC10387210

[64] Zeng Y, Shi L, Liu C, Li W, Li J, Yang S, Yang X, Huang Q, Yang L. Effects of social norm feedback on antibiotic prescribing and its characteristics in behaviour change techniques: a mixed-methods systematic review. Lancet Infect Dis. 2023;23:e175–84. 36521504 10.1016/S1473-3099(22)00720-4

[65] Chenot J-F, Scherer M, Becker A, et al. Acceptance and perceived barriers of implementing a guideline for managing low back in general practice. Implement Sci IS. 2008;3:7. 18257923 10.1186/1748-5908-3-7 PMC2275295

