# Supplementary material for: Self-reported acceptability and feasibility of a multimodal intervention to reduce antibiotic prescriptions for urinary tract infections in primary care: a process evaluation of the RedAres trial among general practitioners and medical practice assistants
Source: BMC Health Serv Res. 2025 Aug 30;25:1160. doi: 10.1186/s12913-025-13218-2 (PMC12399011; doi:10.1186/s12913-025-13218-2)
Supplement: Supplementary file 1 — Supplementary Material 1 [file 12913_2025_13218_MOESM1_ESM.docx]

Process evaluation t2 – final Evaluation

Final visit intervention practice: **GPs** Practice-ID: _____________

| **How much do you agree with the following statements** | | | | | | |
| --- | --- | --- | --- | --- | --- | --- |
| SE_ self-efficacy; AA_affective attitude; PE_ perceived effectiveness; IG_information gain | | | | | | |
|  | Strongly agree |  |  |  | Strongly disagree | n.s. |
| **Prescription feedback:** |  |  |  |  |  |  |
| PF 1) The RedAres prescription feedback had a positive impact on my prescribing behavior (SE) | O | O | O | O | O | O |
| PF 2) I was able to accept the RedAres prescription feedback in relation to my prescribing behavior (AA) | O | O | O | O | O | O |
| PF 3) The RedAres prescription feedback was a valuable resource for me in my consultations, allowing me to effectively refine my prescribing practices. (PE). | O | O | O | O | O | O |
| **Benchmarking:** |  |  |  |  |  |  |
| B 1) The comparison of my prescribing behavior with that in other practices had a positive impact on my prescribing behavior (SE). | O | O | O | O | O | O |
| B 2) I was able to accept the direct comparison of my prescribing behavior with that of other practices (AA). | O | O | O | O | O | O |
| B 3) The RedAres benchmarking was a valuable resource for me in my consultations, allowing me to effectively refine my prescribing practices. (PE). | O | O | O | O | O | O |
| **Resistence data:** |  |  |  |  |  |  |
| R 1) Up to data information on local resistance data had a positive impact on my prescribing behavior (SE) | O | O | O | O | O | O |
| R 2) I was able to accept the up to data information on local resistance data (AA) | O | O | O | O | O | O |
| R 3) The up to data information on local resistance data was a valuable resource for me in my consultations, allowing me to effectively refine my prescribing practices (PE) | O | O | O | O | O | O |
| R 4) Through the up to data information on local resistance I have gained new insights (IG). | O | O | O | O | O | O |
|  |  |  |  |  |  |  |
| **Pocket card & Guideline** | Strongly agree |  |  |  | Strongly disagree |  |
| PG 1) The information materials on guideline-adherent treatment had a positive impact on my prescribing behavior (SE). | O | O | O | O | O | O |
| PG 2): I was able to accept the information materials on guideline-adherent treatment (AA) | O | O | O | O | O | O |
| PG 3) The information materials on guideline-adherent treatment were a valuable resource for me in my consultations, allowing me to effectively refine my prescribing practices (PE) | O | O | O | O | O | O |
| PG 4) Through the information materials on guideline-adherent treatment I have gained new insights (IG) | O | O | O | O | O | O |
| **General** |  |  |  |  |  |  |
| G 1) I believe that the RedAres intervention, a combining personalized feedback, benchmarking, information on resistance data, and informative materials, is well-positioned to positively influence prescribing habits | O | O | O | O | O | O |
| G 2) Which components of the intervention to enhance guideline adherence did you find most helpful? (Multiple answers are possible) | O Prescription feedback O Benchmarking with other practices  O Information on antibiotic resistances  O Pocket card O Guideline | | | | |  |
| G 3) In which form do you prefer information materials in the context of general medical research projects in your practice? | O Print (e.g. flyer, brochure, poster) O Digital (e.g. websites, E-Mail) O _______________________________________ | | | | |  |
| G 4) Have you participated to general medicine research with your practice in the past? | O yes, several times  O yes, once  O no  O comments: | | | | |  |
| G 5) Could you imagine supporting another general medicine research project? | O yes O yes, but ________________________________ O no  Comments: ____________________________  Topic: __________________________________ | | | | |  |
| G 6) Are you interested in participating to a regional practice network? | O yes, I´m already part of a network  O yes, I´m thinking about it*  O no  *Deliver flyer and eventually note email address | | | | |  |
