# Supplementary material for: Self-reported acceptability and feasibility of a multimodal intervention to reduce antibiotic prescriptions for urinary tract infections in primary care: a process evaluation of the RedAres trial among general practitioners and medical practice assistants
Source: BMC Health Serv Res. 2025 Aug 30;25:1160. doi: 10.1186/s12913-025-13218-2 (PMC12399011; doi:10.1186/s12913-025-13218-2)
Supplement: Supplementary file 2 — Supplementary Material 2 [file 12913_2025_13218_MOESM2_ESM.docx]

Process evaluation t2 – final evaluation

Final visit intervention practice: **MPA** Practice-ID: _____________

| **How satisfied are you with the RedAres data collection, documentation, and transmission in your practice in general?**  **How difficult do you consider the following steps of data collection?**   1. Filter data in electronic medical record 2. Extract data by filling the case form 3. Aggregate extracted data with tally sheet 4. Transfer to data extraction form 5. Transfer into REDCap database | (please check)   \| ☺ \|  \|  \| ☹ \| n.s.* \| \| --- \| --- \| --- \| --- \| --- \| \| O \| O \| O \| O \| O \| \| very satisfied \| rather satisfied \| rather unsatified \| very unsatified \|  \|  \| Very  easy \| rather easy \| rather difficult \| Very difficult \| n.s.* \| \| --- \| --- \| --- \| --- \| --- \| \|  \|  \|  \|  \|  \| \| O \| O \| O \| O \| O \| \|  \|  \|  \|  \|  \| \| O \| O \| O \| O \| O \| \|  \|  \|  \|  \|  \| \|  \|  \|  \|  \|  \| \| O \| O \| O \| O \| O \| \|  \|  \|  \|  \|  \| \|  \|  \|  \|  \|  \| \| O \| O \| O \| O \| O \| \|  \|  \|  \|  \|  \| \| O \| O \| O \| O \| O \|   *e.g. does not apply; cannot assess |
| --- | --- | --- | --- | --- | --- | --- | --- | --- | --- | --- | --- | --- | --- | --- | --- | --- | --- | --- | --- | --- | --- | --- | --- | --- | --- | --- | --- | --- | --- | --- | --- | --- | --- | --- | --- | --- | --- | --- | --- | --- | --- | --- | --- | --- | --- | --- | --- | --- | --- | --- | --- | --- | --- | --- | --- | --- | --- | --- | --- | --- | --- | --- | --- | --- | --- | --- | --- | --- | --- | --- | --- | --- | --- | --- | --- | --- | --- | --- | --- | --- | --- |
| The following motivated me to participate to the RedAres study: | O financial incentive O to learn something new O to be part of a research project O to do something else the usual working routines O nothing of the above  O _____________________________________ |
| What went well? What should we keep (in a similar form)? |  |
| What could we do differently/improve? What would you wish for in the future? |  |
| Have you participated to general medicine research with your practice in the past or have you gained research experiences in other contexts? | O yes, several times  O yes, once O no  comments: ____________________________ |
| Do you feel that your skills in data collection and management for studies have improved through your participation in the RedAres study? | O yes O yes, a little O no |
| If yes: Have you been able to use the skills you have learnt for other activities in practice? | O yes Which ones? _______________________________  O no |
| Do you feel that your competences are better perceived by the practice team as a result of taking responsibility for data collection and management in RedAres study? | O yes O yes, a little  O no  comments: ____________________________ |
| How confident do you feel about mastering data collection and administration in another general medical study? | \| Very  confident \| Rather confident \| Rather  unconfident \| very unconfident \| n.s.* \| \| --- \| --- \| --- \| --- \| --- \| \| O \| O \| O \| O \| O \|   *e.g. does not apply; cannot assess |
| Could you imagine taking part in RedAres again? | O yes O yes, but______________________________ O no  comments: ____________________________ |
| Could you imagine supporting another general medical research project? | O yes O yes, but ________________________________ O no  comments: ____________________________  topic: _________________________________ |
