# Supplementary material for: Self-reported acceptability and feasibility of a multimodal intervention to reduce antibiotic prescriptions for urinary tract infections in primary care: a process evaluation of the RedAres trial among general practitioners and medical practice assistants
Source: BMC Health Serv Res. 2025 Aug 30;25:1160. doi: 10.1186/s12913-025-13218-2 (PMC12399011; doi:10.1186/s12913-025-13218-2)
Supplement: Supplementary file 3 — Supplementary Material 3 [file 12913_2025_13218_MOESM3_ESM.docx]

| **1. Basic data** | | |
| --- | --- | --- |
| Practice-ID: | Year of birth____________ | Gender ❑ m ❑ f ❑ d |
| **2.**  **Professional experience/position** | | |
| Number of years in the profession |  |  |
| ❒ ≤ 5 years | ❒ 6-15 years | ❒ ≥ 15 years |
| Position in practice: |  |  |
| ❒ owner | ❒ employed |  |
| **3.**  **Questions about the practice** | | |
| How many inhabitants are there in the town where you work or where your practice is located?  *If you work in more than one place, please refer to the main place where you work.* | | |
| ❒ < 5.000 | ❒ 5.000 - <20.000 | ❒ 20.000 - <100.000 |
| ❒ > 100.000 | ❑ >300.000 | ❑ > 500.000 |
| Weekly working hours | | |
| ❒ full time | ❒ part time  *If part-time, how many hours per week?* | _ _ _ _ _ _ _ _ hours /week |

**Sociodemographic questionnaire general practitioners**

| Your practice is a: (multiple answers are possible) | | | | | |
| --- | --- | --- | --- | --- | --- |
| ❒ single handed practice | | ❒ single handed practice with several doctors under one roof | | ❒ group practice | |
| ❒ teaching practice | | ❒ practice involved in resident training | |  | |
| How many accreditations with the statutory health insurance do you have? Please note ❒ ________________ ❒ n.s. | | | | | |
| Average number of consultations for the entire practice per quarter | | | | | |
| ❒ < 500 | ❒ 500 – < 1.000 | | ❒ 1.000 – < 1.500 | | ❒ > 1.500 |
| How many doctors are employed in your practice? | | | | | |
| Please enter the number of full-time positions. For example: "1.5" for one 100% and one 50% position, 1 for two 50% positions, etc.) | | | _ _ _ _ _ _ _ _ number of employments | | |
