# Supplementary material for: Self-reported acceptability and feasibility of a multimodal intervention to reduce antibiotic prescriptions for urinary tract infections in primary care: a process evaluation of the RedAres trial among general practitioners and medical practice assistants
Source: BMC Health Serv Res. 2025 Aug 30;25:1160. doi: 10.1186/s12913-025-13218-2 (PMC12399011; doi:10.1186/s12913-025-13218-2)
Supplement: Supplementary file 4 — Supplementary Material 4 [file 12913_2025_13218_MOESM4_ESM.docx]

| Practice-ID: |  |
| --- | --- |

Process evaluation t1 medical practice assistant

| Questionnaire personal and practice details | | | |
| --- | --- | --- | --- |
| E01 | Gender: | 🞏_1_ male 🞏_2_ female 🞏_3_ divers | |
| E02 | Which training have you received that is directly applicable to your current work at the practice?"***?*** *(multiple answers are possible)* | | 🞏_1_ medical assistant |
|  |  |  | 🞏_2 (_Paediatric) nurse, nursing specialist (or similar) |
|  |  |  | 🞏_3_ nursing assistant |
|  |  |  | 🞏_4_ other training, namely: ______________________________________ |
|  |  |  | 🞏_5_ none |
| E03 | What (additional) qualifications do you have in relation to your current job? *(multiple answers are possible)* | | 🞏_1_ Non-medical practice assistants |
|  |  |  | 🞏_2_ Care assistant in the GP practice |
|  |  |  | 🞏_3_ practice manager |
|  |  |  | 🞏_4_ other qualification, namely: _____________________________________ |
|  |  |  | 🞏_5_ none |
| E04 | How many years have you been working in your current occupation? (not necessarily in your current practice) | | |
|  | ______ years | | |
| E05 | How many hours per week do you work on average in the practice (in relation to all practice activities)? | | |
|  | 🞏_1_ < 20 🞏_2_ 20 - < 30 🞏_3_ 30 - < 40 🞏_4_ > 40 | | |

**Thank you for your participation!**
