# Supplementary material for: Self-reported acceptability and feasibility of a multimodal intervention to reduce antibiotic prescriptions for urinary tract infections in primary care: a process evaluation of the RedAres trial among general practitioners and medical practice assistants
Source: BMC Health Serv Res. 2025 Aug 30;25:1160. doi: 10.1186/s12913-025-13218-2 (PMC12399011; doi:10.1186/s12913-025-13218-2)
Supplement: Supplementary file 5 — Supplementary Material 5 [file 12913_2025_13218_MOESM5_ESM.docx]

## Supplement 5 Stratified practice characteristics of GPs participating in the RedAres study

| **n/N**  **N=63*** | **n (%)*** | **Gender**  **M (n (%))**  **W (n (%))** | | **Age group**  **<42 (n (%))/43-52 (n (%))/**  **53-62 (n (%))/>63 (n (%))** | **Federal region**  **BER (n (%))/BW (n (%))/**  **BY (n (%))/TH (n (%))** | **Position in practice**  **Self-employed (n (%))/**  **employed (n (%))** | **Working hours**  **VZ (n (%))/**  **TZ (n (%))** | **Years of experience in practice**  **<5 (n (%))/ 6-15 (n (%))/**  **>15 (n (%))** |
| --- | --- | --- | --- | --- | --- | --- | --- | --- |
| doctors employed (57/63) Median (IQR) | 2.0 (1.0-3.0) | 2.2 (1.0-3.4)  1.6 (1.0-3.1) | | 1.9 (1.3-3.5) / 1.0 (1.0-2.8) /  2.0 (1.0-3.0) / 2.8 (1.8-3.6) | 2.9 (1.0-3.3) /3.0 (1.5-3.8) / 1.0 (1.0-2.5) /  1.7 (0.5-2.0) | 1.7 (1.0-3.0) /  2.9 (1.8-3.9) | 2.0 (1.0-3.0) /  1.9 (1.4-2.1) | 2.0 (1.5-3.7) /1.6 (1.0-3.0) /  2.0 (1.0-3.0) |
| Practice type (57/63) | | | | | | | | |
| Single practice | 34 (59.7) | 19 (55.9)  15 (44.1) | | 6 (17.6) / 14 (41.2) /  13 (38.2) / 1 (2.9) | 10 (29.4) / 4 (11.8) /  13 (38.2) / 7 (20.6) | 29 (85.3) /  5 (14.7) | 30 (88.2) /  4 (11.8) | 4 (11.8) / 10 (29.4) /  20 (58.8) |
| group practice | 23 (40.3) | 10 (43.5)  13 (56.5) | | 6 (28.6) / 3 (14.3) /  7 (33.3) / 5 (23.8) | 5 (21,7.0) / 9 (39.1) /  6 (26.0) / 3 (13.1) | 19 (82.6) /  4 (17.4) | 17 (73.9) /  6 (26.1) | 2 (8.7) /8 (34.8) /  13 (56.5) |
| Training practice (57/63) | | | | | | | | |
| Yes | 34(57.9) | 16 (54.5)  18 (45.5) | | 9 (27.3) / 9 (27.3) /  12 (36.4) / 3 (9.1) | 10 (30.3) / 11 (33.3) /  7 (21.2) / 5 (15.1) | 26 (76,5) /  8 (23,5) | 27 (79.4) /  7 (20.6) | 6 (17.6) / 10 (29.4) /  18 (53.0) |
| Number of consultations per quartile (57/63) | | | | | | | | |
| 500-999 | 14 (24.6) | | 5 (35.7) /  9 (64.3) | 2 (14.3) / 6 (42.9) /  5 (35.7) / 1 (7.1) | 3 (21.4) / 3 (21.4) /  6 (42.9) / 2 (14.3) | 13 (92.9) /  1 (7.1) | 11 (78.6) /  22 (91.7) | 0 (0.0) /  5 (35.7) /  9 (64.3) |
| 1000-1499 | 24 (41.2) | | 13 (54.2) /  11 (45.8) | 2 (8.7) / 9 (39.1) /  10 (43.5) / 2 (8.7) | 6 (25.0) / 4 (16.7) /  9 (37.5) / 5 (20.8) | 23 (95.8) /  1 (4.2) | 22 (91.7) /  2 (8.3) | 1 (4.2) /  7 (29.2) /  16 (66.7) |
| >1500 | 19 (34.2) | | 11 (57.9) /  8 (42.1) | 8 (42.1) / 3 (15.8) /  5 (26.3) / 3 (15.8) | 6 (31.6) / 6 (31.6) /  4 (21.1) / 3 (15.8) | 13 (65.0) /  7 (35.0) | 15 (75.0) /  5 (25.0) | 5 (25.0) /  6 (30.0) /  9 (45.0) |
| Number of inhabitants in practice location (57/63) | | | | | | | | |
| <5000 | 10 (17.5) | | 5 (50.0) /  5 (50.0) | 4 (40.0) / 1 (10.0) /  4 (40.0) / 1 (10.0) | 0 (0.0) / 4 (40.0) /  6 (60.0) / 0 (0.0) | 9 (90.0) /  1 (10.0) | 9 (90.0) /  1 (10.0) | 1 (10.0) /3 (30.0) /6 (60.0) |
| 5000-19999 | 21 (36.8) | | 11 (50.0) /  11 (50.0) | 4 (20.0) / 6 (30.0) /  7 (35.0) / 3 (15.0) | 3 (14.3) / 5 (23.8) /  7 (33.3) / 6 (28.6) | 18 (85.7) /  3 (14.3) | 18 (85.7) /  3 (14.3) | 3 (13.6) /6 (27.3) /13 (59.1) |
| 20000-99999 | 10 (17.5) | | 3 (30.0) /  7 (70.0) | 1 (10.0) / 5 (50.0) /  3 (30.0) / 1 (10.0) | 2 (20.0) / 3 (30.0) /  3 (30.0) / 2 (20.0) | 9 (90.0) /  1 (10.0) | 8 (80.0) /  2 (20.0) | 0 (0.0) /1 (16.7) /5 (83.3) |
| 100000-299999 | 6 (10.5) | | 4 (66.7) /  2 (33.3) | 0 (0.0) / 3 (50.0) /  3 (50.0) / 0 (0.0) | 0 (0.0) / 1 (16.7) /  3 (50.0) / 2 (33.3) | 6 (100.0) /  0 (0.0) | 4 (66.7) /  2 (33.3) | 0 (0.0) /1 (16.7) /5 (83.3) |
| >300000 | 10 (17.5) | | 6 (60.0) /  4 (40.0) | 3 (33.3) / 2 (22.2) /  3 (33.3) / 1 (11.1) | 10 (100.0) / 0 (0.0) /  0 (0.0) / 0 (0.0) | 6 (60.0) /  4 (40.0) | 8 (80.0) /  2 (20.0) | 2 (20.0) /3 (30.0) /5 (50.0) |

* **Weighted according to replays per practice (N=57)**
